# Bidirectional Relationship Between Sleep Disturbances and Parkinson's Disease

**DOI:** 10.3389/fneur.2022.927994

**Published:** 2022-07-18

**Authors:** Eiko N. Minakawa

**Affiliations:** ^1^Department of Neurophysiology, National Institute of Neuroscience, National Center of Neurology and Psychiatry, Kodaira, Japan; ^2^Parkinson Disease and Movement Disorder Center, National Center Hospital, National Center of Neurology and Psychiatry, Kodaira, Japan; ^3^Sleep Disorder Center, National Center Hospital, National Center of Neurology and Psychiatry, Kodaira, Japan; ^4^Research Center for Neurocognitive Disorders, National Center Hospital, National Center of Neurology and Psychiatry, Kodaira, Japan

**Keywords:** Parkinson's disease, α-synuclein, sleep disturbances, insomnia, sleep fragmentation, bidirectional relationship

## Abstract

Parkinson's disease (PD) is the second most common neurodegenerative disease after Alzheimer's disease (AD). Both diseases share common clinical and pathological features: the gradual progression of neurological and psychiatric symptoms caused by neuronal dysfunction and neuronal cell death due to the accumulation of misfolded and neurotoxic proteins. Furthermore, both of them are multifactorial diseases in which both genetic and non-genetic factors contribute to the disease course. Non-genetic factors are of particular interest for the development of preventive and therapeutic approaches for these diseases because they are modifiable; of these, sleep is a particularly intriguing factor. Sleep disturbances are highly prevalent among both patients with AD and PD. To date, research has suggested that sleep disturbances are a consequence as well as a risk factor for the onset and progression of AD, which implies a bidirectional relationship between sleep and AD. Whether such a relationship exists in PD is less certain, albeit highly plausible given the shared pathomechanisms. This review examines the current evidence for the bidirectional relationship between sleep and PD. It includes research in both humans and animal models, followed by a discussion of the current understanding of the mechanisms underlying this relationship. Finally, potential avenues of research toward achieving disease modification to treat or prevent PD are proposed. Although further efforts are crucial for preventing the onset and slowing the progress of PD, it is evident that sleep is a valuable candidate target for future interventions to improve the outcomes of PD patients.

## Introduction

Parkinson's disease (PD) is the second most common neurodegenerative disease after Alzheimer's disease (AD) (1). The core clinical features of PD are a gradual progression of motor symptoms, such as bradykinesia, rest tremor, rigidity, and postural instability. In addition, most patients experience a variety of non-motor symptoms, such as sleep dysfunction, autonomic dysfunction, and psychiatric dysfunction (2), which affect their quality of life (3). Despite a global prevalence of more than 6 million individuals and a 2.5-times increase in PD prevalence over the past 30 years (1, 4), current treatments for PD remain limited to symptomatic therapies, such as dopamine replacement, and there is a continued unmet clinical need for disease modification for PD (5).

PD and AD share common clinical and pathological features of neurodegenerative diseases: the slow progression of neurological and psychiatric symptoms caused by the progressive degeneration of neurons in selective central nervous system regions that are characteristic of each disease (6). Furthermore, a common core mechanism underlying both diseases is the accumulation of misfolded and aggregation-prone neurotoxic proteins, such as α-synuclein (α-syn) in PD and amyloid β (Aβ) and tau in AD (6).

PD and AD are multifactorial diseases in which various genetic and non-genetic factors contribute to the disease course (5, 7). Although genetic factors are difficult to modify following birth, non-genetic factors are modifiable and may serve as preventive or therapeutic targets. Of such non-genetic disease-modifiable factors, sleep has attracted attention as a particularly strong candidate for PD and AD (8).

Sleep disturbances are highly prevalent among patients with PD and AD and often appears from the early or prodromal stages of both diseases (8, 9). Sleep disturbances among patients with AD are primarily attributed to AD pathology in brain regions that regulate the sleep-wake or circadian rhythm, such as galaninergic neurons in the intermediate nucleus of the hypothalamus (10), the cholinergic neuronal network that comprises the brainstem, thalamus, basal forebrain, and cerebral cortex (11), and the hypothalamic suprachiasmatic nucleus (12–14). Recently, a novel concept of a bidirectional relationship between AD and sleep disturbances has emerged; in addition to the conventional concept that sleep disturbance is a consequence of AD pathology, epidemiological and experimental evidence has suggested that sleep disturbances are a risk factor for the onset and progression of AD (15). In light of this, alongside the shared pathomechanisms of AD and PD, epidemiological and experimental explorations on whether such a bidirectional relationship exists between PD and impaired sleep are now being conducted.

This review first provides an overview of sleep disturbances in patients with PD and summarizes recent studies that have suggested a bidirectional relationship between PD and sleep disturbances. The potential mechanisms underlying the link between sleep disturbances and PD are then described. Finally, future research directions that could aid in the development of novel therapeutic and preventive methods to alter the disease course of PD by targeting sleep are discussed.

## Sleep Disturbances in PD

Sleep disturbances are one of the most common non-motor symptoms of PD and affect 60–98% of patients with PD (16). Patients with PD experience a broad range of sleep disorders, such as insomnia, excessive daytime somnolence (EDS), sleep-related breathing disorders (SBD), circadian disorders, sleep-related movement disorders [e.g., restless legs syndrome (RLS) and periodic leg movements of sleep (PLMS)], and parasomnia [e.g., rapid eye movement (REM) sleep behavior disorder (RBD)] (16). Among these, insomnia is the most common and affects up to 80% of all PD patients (17). Symptoms of insomnia comprise difficulties initiating sleep, difficulties maintaining sleep, or waking up earlier than desired. Patients with PD usually do not experience significant difficulties in initiating sleep but are unable to maintain sleep (18, 19). Indeed, 81.54% [95% confidence interval (CI) 78.5–84.4] of PD patients reported difficulty in maintaining sleep, whereas 18.0% (95% CI 15.1–20.9) reported difficulty in initiating sleep (17). Thus, sleep fragmentation, a key indicator of sleep maintenance insomnia, is the most common sleep complaint among patients with PD (20).

Insomnia is also one of the most common sleep disturbances among older adults without PD (21). Similar to patients with PD, difficulty in maintaining sleep is the most prevalent symptom among insomnia patients without PD (~50–70%) (22). Although advanced age is a significant risk factor for both PD and insomnia (5, 23), the prevalence of insomnia in patients with PD is higher than that in otherwise healthy older adults (17, 22).

Sleep disturbances are assessed using subjective measures, such as self-report questionnaires, or objective measures, such as polysomnography (PSG) or actigraphy. PSG is useful for distinguishing two major components of sleep, namely REM sleep and non-REM (NREM) sleep, and also distinguishing NREM sleep stages, namely stages N1, N2, and N3 (24). Stage N3, the deepest NREM sleep stage, is defined as a sleep epoch containing 20% or more high-voltage slow waves with a frequency of 0.5–2 Hz and peak-to-peak amplitude > 75 μV as measured by electroencephalogram and is thus referred to as slow-wave sleep (SWS) (25). A recent meta-analysis of PSG studies in patients with PD revealed significant reductions in total sleep time, sleep efficiency, proportion of stage N2 sleep, SWS, and REM sleep in PD patients compared with controls (26). Significant increases in wake time after sleep onset (WASO), proportion of stage N1 sleep, and REM latency have also been observed in patients with PD (26). Intriguingly, a supplementary meta-analysis revealed no significant differences in PSG parameters that directly measure sleep architecture related to sleep maintenance, such as WASO, sleep efficiency, and N1, N2, and SWS percentages, between PD patients with and without RBD, which is a major sleep disorder experienced by patients with prodromal PD (26). However, this analysis found differences in arousal index, PLMS index, and REM percentages, which could affect the sleep architecture (26).

Sleep disturbances are often under-reported by patients with PD and under-diagnosed by health professionals (27). One reason for this under-recognition may be the occasional discrepancy between the subjective complaints of sleep problems and the objective abnormality of sleep structure measured using PSG (28, 29). Notably, although objective sleep abnormalities may be subtle in otherwise healthy people with subjective sleep disturbances, there are often significant objective sleep abnormalities in addition to subjective sleep disturbances in patients with PD (30). A recent study found that, despite a significant association between subjective sleep quality and health-related quality of life (HRQoL), the associations between objective measurements of PSG and HRQoL were negligible, indicating that objective sleep abnormalities may not be perceived as sleep disturbances in patients with PD (31). Such discrepancies that are characteristic of patients with PD could contribute to the under-recognition of sleep disturbances despite their high prevalence and severity.

## Bidirectional Relationship Between Sleep and PD

Sleep disturbance in PD is multifactorial (32) in contrast with that in AD, which is mainly due to the AD pathology affecting the brain regions regulating sleep-wake or circadian rhythm (8, 10–14). Both dopaminergic and various non-dopaminergic degeneration in PD alters the regulation of sleep-wake and circadian rhythm (33). Furthermore, numerous other factors contribute to insomnia in PD patients, often in combination: nocturnal motor symptoms such as hypokinesia or dystonia, non-motor symptoms such as nocturia or pain, mental comorbidities such as depression or anxiety, and sleep problems other than insomnia such as SBD, RLS, and PLMS (33). Moreover, dopaminergic medication for PD can induce sleep disturbances, such as insomnia and EDS (34), although long-acting dopamine agonists modestly improve insomnia symptoms possibly via reducing nocturnal motor and some non-motor symptoms (35–37).

**Figure 1:**
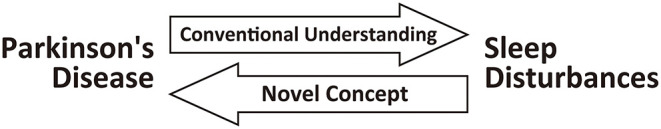
Bidirectional relationship between sleep disturbances and Parkinson's disease (PD). Sleep disturbances, especially insomnia, are one of the most common non-motor symptoms in patients with PD and otherwise healthy older adults. The conventional understanding is that PD pathology and various PD-related motor or non-motor symptoms induce sleep disturbances in patients with PD (upper arrow). In contrast, recent epidemiological and experimental studies have suggested that sleep disturbances impact the susceptibility to developing or the progression of PD (lower arrow). These are not mutually exclusive and could form a vicious cycle in which sleep disturbances caused by PD pathology in turn accelerate the PD pathology.

These observations suggest that sleep disturbances are a consequence of PD progression (Figure 1; upper arrow). However, recent studies have investigated the impact of sleep disturbances on the susceptibility to developing or the progression of PD. A retrospective longitudinal cohort study conducted in Taiwan using a nationwide database revealed that non-apnea sleep disorders, especially insomnia, are associated with a significantly higher risk of future development of PD (38). In a cohort of 91,273 adult patients with non-apnea sleep disorders without PD and a control cohort, non-apnea sleep disorders were found to be an independent risk factor for future development of PD after multivariate adjustment (adjusted hazard ratio: 1.18, 95% CI: 1.11–1.26, *p* < 0.001) (34). Importantly, among the various non-apnea sleep disorders, patients with chronic insomnia lasting for more than 3 months have the greatest risk for future development of PD (adjusted hazard ratio: 1.37, 95% CI: 1.21–1.55, *p* < 0.001) (38). Another large prospective longitudinal population-based study with over 64,855 person-years and 13 years of follow-up used subjective sleep assessment and demonstrated that subjectively poorer sleep quality and shorter sleep duration are associated with an increased risk of incident Parkinsonism within the first 2 years of follow-up, which attenuated during follow-up (39). This is in line with a large registry-based study that showed increases in insomnia diagnoses 2 years but not 5 and 10 years before diagnosis of PD (40). Although these two studies suggest that sleep disturbances are prodromal or early symptoms of PD, they do not exclude the possibility that sleep disturbances may simultaneously accelerate the underlying PD pathology.

In addition, greater sleep fragmentation measured by actigraphy in older adults without a clinical diagnosis of PD is associated with higher odds of subclinical PD pathology, such as the presence of Lewy bodies [LBs; odds ratio (OR): 1.40, 95% CI: 1.05–1.86, *p* = 0.02] and substantia nigra neuronal loss (OR: 1.43, 95% CI: 1.10–1.88, *p* = 0.008), and a pathological diagnosis of PD (OR: 2.04, 95% CI: 1.34–3.16, *p* = 0.0009) (41). Importantly, these associations were independent of various medical and psychiatric comorbidities, including cognitive impairment and AD pathology (41). Furthermore, in a retrospective longitudinal study in 129 patients with PD who underwent PSG followed by a regular clinical assessment including the Unified Parkinson's Disease Rating Scale Part III for at least 2 years, lower accumulated power of SWS was shown to be associated with faster motor symptom progression, especially axial motor symptoms, over an average follow-up period of 4.6 ± 2.3 years (42).

All of the abovementioned clinical studies have demonstrated the association between impaired sleep and increased risk of PD onset or progression and thus suggest a bidirectional relationship between sleep disturbances and PD (Figure 1). One interpretation of these results is that sleep disturbances are a prodromal symptom of PD caused by early PD pathology that affects the sleep regulatory circuit or circadian rhythm, which is well established in the case of RBD and also likely in the case of insomnia based on the clinicoepidemiological studies described above. An alternative explanation is that sleep disturbances accelerate the onset and progression of PD. The subtype(s) of sleep disturbances or the component(s) of sleep that could affect PD pathology needs to be determined in future studies. These two mechanisms are not mutually exclusive and could form a vicious cycle in which sleep disturbances caused by PD pathology in turn accelerate the PD pathology.

Further investigation of this bidirectional relationship between sleep disturbances and PD, especially the precise causal relationship, is vital to determine whether treating sleep disturbances in the general population and patients with PD would help prevent or delay the onset and progression of PD. Various animal models of PD, especially mouse models, are useful for examining the causal relationship between sleep and PD because the core mechanisms underlying the sleep-wake cycle and its regulation are shared between humans and mice (43). A recent study demonstrated in mouse models of PD that the administration of sodium oxybate enhanced SWS and reduced the accumulation of aggregated α-syn in the brain, although no major symptomatic effects were observed (44). Further investigations of the causal relationship between sleep disturbances and PD with a particular focus on PD symptoms and pathology are needed.

## Possible Mechanisms Underlying the Bidirectional Relationship Between Sleep and PD

Homeostatic regulation of protein quality, referred to as proteostasis, is indispensable for human health (45). Proteostasis is maintained via the coordination of multiple intra- and extracellular systems that regulate protein synthesis, folding, disaggregation, and degradation or elimination (45). Impaired proteostasis and the resulting accumulation of misfolded and aggregation-prone neurotoxic proteins, including α-syn in PD and Aβ and tau in AD, are common pathomechanisms that underlie neurodegenerative diseases (6). Various studies have demonstrated that sleep contributes to the dynamics of these neurotoxic proteins, although studies on α-syn dynamics in relation to sleep are still in their infancy (46).

In AD mice, the two pathological hallmarks of AD, namely extracellular senile plaques composed mainly of Aβ and intracellular neurofibrillary tangles (NFTs) composed mainly of phosphorylated tau, increase with sleep restriction or sleep fragmentation (47–51). An increase in neuronal activity, such as chemical or optogenetic stimulation in mice or sustained wakefulness due to sleep restriction in both mice and humans, raises the extracellular secretion of soluble Aβ (52–56), which leads to the extracellular accumulation of aggregated insoluble Aβ (as demonstrated in mice) (47, 48, 57) because Aβ has a high propensity to aggregate in a concentration-dependent manner (58). Moreover, an increase in neuronal activity, including sustained wakefulness, increases extracellular tau and modifies its phosphorylation *in vivo* (49, 59–62). However, exactly how this extracellular secretion of tau relates to intracellular tau accumulation and NFT formation remains to be elucidated. One plausible mechanism is the cell-to-cell propagation of tau pathology, which is the spreading of tau pathology via extracellular tau secretion followed by the uptake of tau by adjacent cells (63).

In addition to the increased secretion of Aβ and tau due to increased neuronal activity, a decrease in clearance efficiency of extracellular metabolites due to poor sleep may contribute to Aβ and tau accumulation. The glymphatic system is a recently proposed mechanism that removes extracellular metabolites, including Aβ, via the interchanging convective flow of interstitial fluid (ISF) and cerebrospinal fluid (CSF) in the brain (64). Aquaporin 4, a water-specific channel protein expressed on the astrocytic endfeet, is considered a key molecule that enables this convective flow (64). The clearance efficiency of extracellular metabolites, including Aβ, via the glymphatic system has been reported to increase in mice by 60% during natural sleep (65). Thus, Nedergaard and colleagues proposed that the physiological restorative function of sleep is the removal of extracellular waste produced during wakefulness (65). However, the existence of the glymphatic system and its relationship with impaired sleep and neurodegenerative diseases remains controversial (66–68).

α-syn is the main component of LBs, the pathological hallmark of PD, and is a neuronal protein that is predominantly found as soluble monomers in the cytoplasm (69). α-syn undergoes conformational changes under specific conditions, which leads to the formation of soluble yet neurotoxic oligomers and protofibrils, followed by the formation of insoluble aggregates found in LBs (70). Similar to Aβ and tau, an increase in neuronal activity in mice and humans, including sustained wakefulness due to sleep restriction, raises the extracellular soluble α-syn in the ISF or CSF (49, 71). In addition, like tau, the cell-to-cell transmission of α-syn has been demonstrated in various *in vitro* and *in vivo* model systems and is suggested to contribute to the progression of α-syn pathology in patients with PD (72). Research on the contribution of extracellular clearance pathways, including the glymphatic system, to the removal of extracellular α-syn is emerging. Zou et al. demonstrated in a mouse model of PD that ligating deep cervical lymph nodes decreases meningeal lymphatic drainage and aggravates α-syn pathology and motor impairment (73). More recently, Ding et al. showed that meningeal lymphatic drainage is significantly decreased in patients with PD (74). However, it is worth noting that the decrease in intracellular α-syn degradation, in addition to the decrease in extracellular α-syn clearance, likely contributed to the aggravation of α-syn pathology and motor phenotype in the PD mice subjected to meningeal lymphatic drainage blockage (73). Additional studies exploring the interplay between sleep and intra- and extracellular α-syn dynamics are necessary to better understand the causal relationship between sleep disturbances and PD.

In addition to the abovementioned potential alterations in extracellular dynamics of neurotoxic proteins due to altered sleep, various intra- and extracellular mechanisms may contribute to impaired proteostasis under sleep disturbances. Sustained wakefulness activates the unfolded protein response pathway, which is one of the core mechanisms that protect cells from the accumulation of neurotoxic proteins (75–78). Aging impairs this protective response against insufficient sleep in mice, and the pro-apoptotic signaling pathways are activated (79). Furthermore, neuroinflammation, blood-brain barrier disruption, oxidative stress, and neuronal double-strand DNA breaks, all of which are known to exacerbate neurodegenerative pathology, are induced by impaired sleep (80–85). Although all of these mechanisms potentially contribute to the common pathomechanism of neurodegenerative diseases, understanding their contribution to α-syn pathology under impaired sleep requires further research.

## Discussion

The number of patients with AD is expected to increase worldwide, particularly in low- and middle-income countries (86). Surprisingly, however, in Europe and North America, the age-specific incidence rate of AD has declined by 13% per decade over the past 25 years (87). Intriguingly, the decrease in the known risk factors of AD during this period does not fully explain the decrease in the incidence of dementia (88). These data highlight the importance of identifying novel modifiable non-genetic factors that could serve as targets for primary and secondary AD prevention. Sleep disturbances have recently emerged as a strong candidate for such novel and modifiable risk factors for AD (8).

It is plausible that sleep disturbances may also serve as a modifiable risk factor for PD (89), given the shared pathomechanisms between AD and PD as neurodegenerative diseases and the common sleep-related changes in the dynamics of neurotoxic proteins involved in AD and PD. On the basis of the heterogeneous spatial and temporal progression patterns of PD symptoms and the underlying pathology, several hypotheses explaining this heterogeneity have been proposed. The Braak hypothesis originally proposed that PD pathology originates in the peripheral autonomic nervous system or in the olfactory bulb and then spreads throughout the brain in a sequential manner (90). Several alternative hypotheses were then proposed to delineate PD subtypes that cannot be explained solely by the Braak hypothesis. For example, Horsager et al. recently hypothesized that PD comprises two subtypes based on progression patterns: brain-first PD and body-first PD (91). In brain-first PD, α-syn pathology arises in the brain and subsequently descends to the peripheral autonomic nervous system via the brainstem (91, 92). In body-first PD, α-syn pathology arises in the peripheral autonomic nervous system and ascends to the brain via autonomic nerves (91, 92). These hypotheses are consistent with the propensity of α-syn to transmit intracellularly along the neuronal circuit in the cellular and animal models of PD and the progression pattern of PD observed in neuropathological and clinical studies (93). However, these hypotheses still remain controversial based on descriptive cliniconeuropathological studies, including an autopsy-based study reporting no cases exhibiting PD pathology in the peripheral nervous system but not in the brain (94–96). Uchihara and Giasson instead proposed a multifocal PD pathology hypothesis based on the observation that PD pathology initially appears in multiple neuronal groups without transsynaptic connections and then spreads into selective but variable neuroanatomical structures (97). Importantly, sleep could serve as a preventive target in either pattern or stage of PD progression, owing to the high prevalence of sleep disturbances among patients with PD and otherwise healthy older adults. In the patients with PD pathology in the brain regions regulating sleep-wake or circadian rhythms, sleep disturbances caused by PD pathology may accelerate PD progression. In other patients, sleep disturbances caused by aging may accelerate PD progression both in the brain and from the peripheral autonomic nervous system into the brain because sleep disturbances evoke the systemic immune response and metabolic stress, both of which are known contributors to PD pathology (98, 99). Furthermore, in both PD subtypes, sleep disturbances due to PD, AD, or aging, may aggravate concomitant AD pathology and/or vascular pathology. Comorbidity of AD pathology is common in PD and affects the clinical phenotype of patients (100–102). Moreover, vascular factors contribute to both AD and PD pathology (58, 103). Consideration of these overlapping and interacting pathologies during the prodromal stages is essential for developing disease-modifying therapeutic and/or preventive methods for PD (104). Sleep is an emerging attractive candidate for such preventive methods that could affect the abovementioned pathologies.

Further clarification of the causal relationship between sleep disturbances and PD in both preclinical and clinical settings is crucial. Complementary approaches by longitudinal observational studies in a large prodromal PD cohort with subjective and objective sleep assessment and by basic studies using animal models of PD that recapitulate both the symptoms and the pathological findings through the prodromal to the motor phase would be helpful. Developing novel methods to monitor the progression of PD pathology *in vivo*, such as α-syn tracer for positron emission tomography, would be a great addition in these studies. Determining the components of “good sleep” that delay or prevent PD progression is also necessary. Transomics analyses of biofluids, such as CSF or plasma, obtained in the aforementioned longitudinal studies in combination with analyses of the sleep profiles and clinical PD progression could help delineate the biological changes underlying sleep-related PD progression. Furthermore, there is an urgent need to develop therapeutic methods to achieve “good sleep.” Non-pharmacological treatments, such as cognitive-behavioral therapy for insomnia (105), may also benefit patients with PD. Meanwhile, novel hypnotics with different mechanisms of action and potentially better safety profiles may offer more suitable therapeutic opportunities for older adults (106). Currently, a phase-2 clinical trial is being conducted to test whether 2-year administration of suvorexant, an orexin receptor antagonist used to treat insomnia, delays Aβ accumulation in the older adults who have mild Aβ accumulation without clinically overt dementia (107). Considering that the number of orexinergic neurons is decreased in patients with PD (108, 109) and that orexin administration partly ameliorates non-motor symptoms in a mouse model of PD (110), whether orexin receptor antagonists as hypnotics are advantageous for patients with PD needs to be carefully determined in future studies. Interdisciplinary collaboration between clinicians and researchers in the various fields described above will contribute to our understanding of the bidirectional relationship between sleep and PD and help develop disease modification methods to overcome the PD pandemic (111, 112).

## Author Contributions

EM contributed to the conception and design of the manuscript, performed the literature search, and wrote the manuscript.

## Funding

This work was funded in part by Grants-in-Aid for Young Scientists (B) (26860681 to EM), Young Scientists (18K15474 to EM), Scientific Research (C) (21K11640 to EM) from the Japan Society for the Promotion of Science Research Grant from Japan Foundation for Neuroscience and Mental Health (to EM), Research Grant from the Naito Foundation (to EM), and Intramural Research Grants for Neurological and Psychiatric Disorders (3-3 to EM) from NCNP, Japan.

## Conflict of Interest

The author declares that the research was conducted in the absence of any commercial or financial relationships that could be construed as a potential conflict of interest.

## Publisher's Note

All claims expressed in this article are solely those of the authors and do not necessarily represent those of their affiliated organizations, or those of the publisher, the editors and the reviewers. Any product that may be evaluated in this article, or claim that may be made by its manufacturer, is not guaranteed or endorsed by the publisher.
